#  Vitamin D receptor expression in sperm of males with unexplained infertility

**DOI:** 10.22088/cjim.14.1.94

**Published:** 2023

**Authors:** Zahra Akbari Sagheb, Mousa Mohammadnia-Afrouzi, Hamid Shafi, Sedigheh Esmailzadeh, Kousar Esmailnejad Ganji, Mehdi Shahbazi

**Affiliations:** 1 Infertility and Reproductive Health Research Center, Health Research Institute, Babol University of Medical Sciences, Babol, Iran; 2 Cellular and Molecular Biology Research Center, Health Research Institute, Babol University of Medical Sciences, Babol, Iran; 3 Immunoregulation Research Center, Health Research Institute, Babol University of Medical Sciences, Babol, Iran

**Keywords:** Vitamin D receptor, Unexplained infertility, Vitamin D, Messenger RNA expression

## Abstract

**Background:**

Vitamin D is associated with numerous disorders, including infertility. Accordingly, the goal of this research was to find out the level of vitamin D and messenger RNA (mRNA) expression of vitamin D receptor (VDR) in the sperm of male subjects with unexplained infertility.

**Methods:**

Twenty-four unexplained infertile men as the case group and 22 healthy fertile men as the control group were recruited. Vitamin D levels were evaluated in seminal fluid using enzyme-linked immunosorbent assay (ELISA). Afterwards, the swim-up test was performed to isolate motile sperm cells. From these cells, RNA was extracted, complementary DNA (cDNA) was synthesized, and mRNA expression of the VDR gene was evaluated with quantitative real-time polymerase chain reaction (PCR).

**Results:**

A decrease in VDR mRNA expression levels was detected in the case group compared to the control group, but this reduction was not statistically significant (p>0.05). Besides, the level of vitamin D in seminal fluid was not detectable in both groups.

**Conclusion:**

The sperm of unexplained infertile men express VDR gene mRNA, although there was no vitamin D in seminal samples. Hence, vitamin D and VDR signaling might not be effective in the etiopathogenesis of unexplained infertility in men.

Today, infertility is a common problem of young couples, defined as a failure to achieve pregnancy after 12 months of unprotected regular intercourse. Male factors are responsible for 50% of all infertility cases. (1). Semen analysis is one of the most used methods for determining the male factor in infertility. Male infertility can cause by several factors, including immunological factors (1, 2). In some infertile patients, no known causes are found for infertility, and all routine tests are normal; this kind of infertility is known as unexplained infertility, which accounts for 10-15% of infertility cases (3, 4). Currently, the significant role of the immune system is clear in reproductive system health. Immune cells and cytokines produced by testis immune cells (or other male reproductive cells) can affect the reproductive system (5, 6). The major role of vitamin D as a steroid hormone is to regulate calcium homeostasis; however, recently, its non-classical function has received a lot of attention, especially immunologic functions (7, 8). Furthermore, it has an important role in the growth and differentiation of many immune cell types (7, 9-11). Vitamin D has been shown to play modulatory roles in the reproductive system (12-14). It exerts its functions via binding to vitamin D receptor (*VDR*), which is a transcription factor and involved in the regulation of gene transcription after ligand-dependent activation (15). Studies have shown that *VDR* and its related metabolizing enzymes are expressed in the male reproductive tract, including germ cells, spermatozoa, Leydig cells, and epithelial cells (16).

A direct effect of vitamin D has been reported on spermatogenesis, sex hormone production, and sperm maturation (17, 18). Numerous studies have indicated a positive correlation between serum levels of vitamin D and sperm motility (19). In addition, *in vitro* assessments of spermatozoa indicated that vitamin D could increase intracellular calcium concentration and sperm motility and induce the acrosome reaction in mature spermatozoa (20). Another report revealed that either high or low serum vitamin D levels could be negatively related to the semen parameters (21, 22). Nevertheless, no study has yet been conducted on evaluating the relationship between vitamin D and unexplained infertility in men.

Seminal fluid has an important role in establishing immune tolerance in the female reproductive tract (23). It consists of components that eventually modulate the immune system (24). Due to the role of vitamin D in the immune system and fertility, this research aimed to evaluate the *VDR* gene expression in sperm samples obtained from unexplained infertile men and compare it with fertile cases. This was, as far as we know, the first study that aimed to identify the role of vitamin D and its receptor on fertility in unexplained infertile men.

## Methods

**Materials/Patients: **The Ethics Committee of Babol University of Medical Sciences (MUBABOL.HRI.REC.1396.27) approved this study. A total of 46 men were selected from those who were referred to the Fatemeh Zahra Infertility and Reproductive Health Research Center, Babol, Iran, for unexplained infertility treatment or normal physical examination from August 2017 to May 2019. Finally, 24 unexplained infertile men as the case group and 22 healthy fertile men as the control group were selected for the final analysis. Informed consent was obtained from patients before sampling. The clinical information and research question was developed according to the World Health Organization (WHO) laboratory manual (sixth edition, 20211) for the examination and processing of human semen, including age, heritable conditions, physical examination, endocrine evaluation, and semen analysis (25). Moreover, in the case group (as a criterion), the wives were of childbearing age without any disorder or abnormality in the reproductive system. Exclusion criteria included men with varicocele, hydrocele, anatomical disorders, scrotal and undescended testis surgery, chemotherapy or radiotherapy, and abnormal semen analysis, as well as wives with reproductive disorders. Thereupon, all the patients without the conditions mentioned above were considered as unexplained infertile cases. Furthermore, 22 healthy fertile men who had at least 1-year-old children been included as the control group; they also had normal fertility and no therapeutic interventions and vitamin D supplementation. Relevant doctors using the obtained information disseminated the results of the study to participants.

**Semen collection and analysis: **Our participants obtained a semen sample by masturbation after 2–5 days of sexual abstinence based on WHO criteria (2021) (25). Semen volume was determined using conical graduated tubes after liquefaction at 37 °C for 20-30 minutes. Sperm concentration, percentage of normal morphology, and motile sperm were evaluated using microscopic examination. In accordance with WHO criteria (2021), sperm/semen samples with normal values for semen volume, sperm count, motility, vitality, and morphology were included in this study. After semen analysis, the samples were divided into 2 portions, 1 portion for biochemical analysis and another for molecular examination.

**Biochemical analysis: **One portion of each sample was used for plasma isolation and measurement of 25-hydroxyvitamin D (25-OHD). First, samples were centrifuged at 8,000 rpm for 10 minutes at 4 °C to separate and remove plasma from the sperm in semen. Seminal plasma was frozen and stored at -80 °C until analysis. Semen levels of 25-OHD were measured using an enzyme-linked immunosorbent assay (ELISA) kit (EUROIMMUN, Germany) on a Rayto Microplate Reader (RT-6100).

**Spiking procedures: **Twenty microliters of semen plasma and 20 µL of 25-OHD standard included in the ELISA kit were combined. Then, known amounts of this solution were used to measure 25-OHD according to the kit’s instructions. 

**Swim-up test for isolation of motile sperm cells: **After liquefaction, motile spermatozoa of each sample was isolated by the swim-up test under the following conditions: The latter portion of samples was combined using the Vita sperm medium (INOCLON, Iran; containing 10% bovine serum albumin (BSA)) in a sterile 15-mL round-bottom centrifuge tube up to 10 mL and gently mixed. Afterwards, the tubes were centrifuged at 2,500 rpm for 10 minutes. Then, the supernatant was separated and discarded without disturbing the pellet, and the pellet was resuspended in 1 mL of fresh medium. Thereafter, the tubes were centrifuged at 2,500 rpm for 5 minutes and incubated at a 45° angle for 30 minutes for swim-up in a 37 °C incubator. After the incubation period, the entire supernatant was aspirated from the pellet and pooled into a sterile 1.5-mL microtube (25). Then, the tubes were centrifuged at 5,500 rpm for 10 minutes, the supernatants were discarded, and the pellets were used for *RNA* extraction.

**RNA extraction, complementary DNA synthesis, and quantitative real-time polymerase chain reaction: **Total *RNA* was isolated from sperm using the RNX-Plus solution (SinaClon, Iran) according to the manufacturer’s instructions. Complementary *DNA* (*cDNA*) was synthesized from sperm *RNA* using a *cDNA* synthesis kit (Yektatajhiz, Iran) according to the manufacturer’s instructions. To determine whether RNA preparations and real-time polymerase chain reactions (PCRs) are free of genomic DNA contamination, PCR was performed with negative controls without *cDNA* (*cDNA*-). PCR was carried out using a PeQlab Thermal Cycler (Germany). Each PCR product was run on 2% agarose gel (containing safe stain SYBR) and photographed under UV light. Primers used in this study were designed using NCBI databases, their quality controlled using the AlleleID software package, and bought from Pishgam Company. Each primer was used at a concentration of 10 µM in each reaction. Quantitative real-time PCR was performed using an Applied Biosystems Step One Plus device and reaction mixtures contained YTA SYBR green qPCR master mix (Yektatajhiz, Iran), *cDNA*, primers for human *VDR* (table 1), and nuclease-free water. The thermocycling conditions were as follows: 2 minutes at 95 °C for initial denaturation, 40 cycles at 95 °C for 20 seconds, 60 °C for 30 seconds, and 72 °C for 30 seconds. Samples were run in duplicate orders. All experiments included negative controls (no *cDNA*).

**Table 1:** Sequence of primers

Product size (bp)	Annealing temperature (°C)	Primer sequence		Gene
**131**	60	5´-CCACCAACCCATCAGAAGGAGAAG-3´	Forward primer	VDR
	60	5´- TGAGGCAACAGCATTATCCAAGGC-3´	Reverse primer	
**228**	60	5'-GAAGGTGAAGGTCGGAGTCAAC -3'	Forward primer	GAPDH
	60	5'-TGGAAGATGGTGATGGGATTTC-3'	Reverse primer	

Abbreviations: *VDR*, vitamin D receptor; *GAPDH*, glyceraldehyde-3-phosphate dehydrogenase; bp, base pair. To calculate the relative messenger *RNA* (*mRNA*) expression of the target gene, the difference in cycle values was determined as the difference between the tested gene and the reference housekeeping gene (glyceraldehyde-3-phosphate dehydrogenase (*GAPDH*)) according to the Livak method (26). The expression level was calculated using the 2^-ΔCT^ formula.

**Statistical analysis: **The results were expressed as mean±SD. Statistical analysis was performed using GraphPad Prism 8 using the independent *t* test. Furthermore, the level of statistical significance was set at p<0.05.

## Results

**Patients:** The mean age was similar between men with unexplained infertility (33±4 years) and healthy donors (34±2 years). Table 2 details the demographic and seminal characteristics of the fertile donors and infertile men. The evaluation of spermatozoa criteria between the 2 groups did not show any significant difference.

**Semen analysis:** All samples had a normal semen analysis, but some minor differences in motility and morphology were seen. Table 2 summarizes the characteristics of semen from the study subjects.

**Biochemical analyses: **To determine the integrity of the methodological approach, we spiked seminal plasma samples with 25-OHD standards, and a normal signal was detected. Data indicated no detections of 25-OHD in seminal plasma from normal and men with unexplained infertility. The detection limit of 25-OHD ELISA was 1.6 ng/mL.

**Table 2:** Clinical information of groups

	Infertile Men	Normal Men
*n*	24	22
**Age, y**	33±4	34±2
**BMI, kg/m** ^2^	22.3±2	21.5±2
**Semen volume, mL**	3.27±2	2.8±1.4
**Total sperm count, 10** ^6^ ** sperm**	273.3	269.1
**Sperm concentration, 10** ^6 ^ **sperm/mL**	86.9±22.7	92.4±22
**Normal morphology, %**	28.2±9.5	41.6±13.9
**Total motility, %**	62.4 ± 7.4	61.1 ± 8.3
**Progressive motility, %**	17	14.6

Abbreviation: BMI, body mass index. Values for infertile and normal men are presented as median (SD).

**Figure 1:**
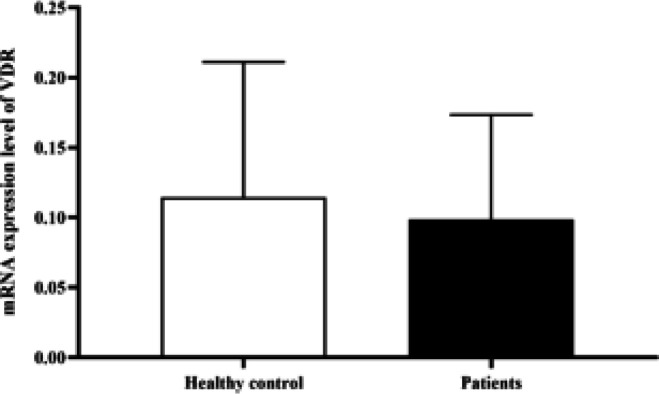
The expression level of VDR gene mRNA in the case and control groups used real-time PCR. The real-time PCR results indicated a statistically insignificant downregulation of VDR mRNA in the case group compared to the control group (P = 0.144)

**Expression of VDR in spermatozoa from infertile men: **As shown in figure 1, the real-time PCR results indicated downregulation of *VDR* gene *mRNA* in the case group compared to the control group, but this difference was not statistically significant (*P *= 0.144).

## Discussion

It has been estimated that approximately 15% of couples suffer from infertility. Previous reports have shown the impact of 1,25 (OH)_2_D_3_ and the *VDR* signaling on the immune system. Otherwise, vitamin D deficiency can modify the reproductive system indirectly by a calcium-mediated mechanism (17, 19, 22, 27). To the best of our knowledge, this is the first study to determine *VDR* gene expression in spermatozoa from unexplained infertile men. 

The baseline characteristics of our preliminary study were first to evaluate seminal levels of vitamin D in the case and control groups, which indicated that semen did not contain vitamin D. This observation is in line with the Bøllehuus Hansen *et al*.’s study, in which measured the seminal vitamin D levels by isotope dilution liquid chromatography–tandem mass spectrometry (LC-MS/MS) (28). In contrast, 1 study could detect vitamin D in semen using an electrochemiluminescence method (29). However, LC-MS is considered the gold standard method for analysis of 25-OHD in biological samples due to the high precision and accuracy versus immunoassay methods (30, 31). Therefore, 1 possible explanation for this discrepancy may be the difference in sensitivity and specificity of the methods used. Thereby, our result could be approved by the first study, which indicated that seminal plasma concentrations of 25-OHD were undetectable. Evidently, to back up and confirm this observation, more researches are needed. Interestingly, we spiked seminal plasma samples with 25-OHD standards, yielding detection of a normal signal, which indicates that the lack of 25-OHD level did not result from suppressive factors of antigen and antibody reactions.

The next part of our result was about *VDR* gene *mRNA* expression level in spermatozoa. Here, in this study, we observed for the first time that the sperm of men with unexplained infertility expressed the *VDR* gene. However, the decrease in *mRNA* expression of the *VDR* gene in unexplained infertile patients was not significantly lower than the fertile group. This observation is in contrast with previous findings on the role of this vitamin and its receptor on sperm function and development and also on fertility, which may stem from a small sample size (32). Previously, studies have described that *VDR* and vitamin D metabolize enzymes in the human ovary, uterus, placenta, testis, prostate, and spermatozoa, suggesting a regulatory role for vitamin D in the reproductive system physiology (33). More recently, studies have shown that vitamin D can increase intracellular calcium concentration through the rapid non-genomic pathway, which induces motility in sperm (17, 34, 35). The high rate of progressive motility in sperm confers easier fertilization and more success and, therefore, can affect reproduction (29). 

Vitamin D exerts its function through *VDR*, and its biological function is associated with a number of *VDR* molecules (36). Additionally, it may induce the anti-inflammatory cytokine production and inhibition of inflammatory cytokine production, thereby playing an immunosuppressive function that leads to the protection of fertilized eggs from an immune attack. Hence, decreased *VDR* can affect fertility through the dysregulated immune system (37, 38). Bøllehuus Hansen *et al*. indicated that the expression levels of *VDR *and CYP24A1 in 114 healthy fertile men were 2-fold higher than 230 infertile men (28). Alternately, Kinuta *et al*.’s animal study demonstrated that *VDR* null mice displayed a substantial decrease in sperm count (40%) and motility (9-fold), which eventually may cause infertility (39). In contrast, some studies have shown that serum vitamin D level does not affect sperm parameters (40, 41). That notwithstanding, no sufficient data are available in unexplained infertility, preventing comparison of our results with previous findings.

Undetectable levels of seminal fluid vitamin D suggest that sperm may expose to vitamin D in the female reproductive tract (29). Furthermore, the presence of vitamin D in the male reproductive tract could result in premature stimulation of the spermatozoa through increasing calcium concentration, which can be another reason for the undetectable concentration of vitamin D in semen. Instead, a high concentration of vitamin D in the follicular fluid may confirm this hypothesis (28). This way, the spermatozoa’s lack of access to vitamin D could be compensated.

In conclusion, this was the first study to evaluate the *mRNA *expression and vitamin D level in cases with unexplained infertility. Although vitamin D was undetectable in seminal fluid, spermatozoa from men expressed *VDR* gene *mRNA*. It can be suggested that signaling of vitamin D and *VDR* might not be involved in the pathogenesis of unexplained infertility. However, further experiments are required to divulge this hypothesis.
